# The Ca^2+^ Sensor Protein Swiprosin-1/EFhd2 Is Present in Neurites and Involved in Kinesin-Mediated Transport in Neurons

**DOI:** 10.1371/journal.pone.0103976

**Published:** 2014-08-18

**Authors:** Pavitra Purohit, Francesc Perez-Branguli, Iryna Prots, Eva Borger, Frank Gunn-Moore, Oliver Welzel, Kristina Loy, Eva Maria Wenzel, Teja W. Grömer, Sebastian Brachs, Max Holzer, Rolf Buslei, Kristin Fritsch, Martin Regensburger, Konrad J. Böhm, Beate Winner, Dirk Mielenz

**Affiliations:** 1 Division of Molecular Immunology, Dept. of Internal Medicine III, Universitätsklinikum Erlangen, and Friedrich-Alexander-Universität Erlangen-Nürnberg, Erlangen, Germany; 2 IZKF Nachwuchsgruppe III, Universitätsklinikum Erlangen, and Friedrich-Alexander-Universität Erlangen-Nürnberg, Erlangen, Germany; 3 School of Biology, Medical and Biological Sciences Building, University of St. Andrews, North Haugh, St Andrews, Fife, United Kingdom; 4 Department of Psychiatry, Friedrich-Alexander-Universität Erlangen-Nürnberg, Erlangen, Germany; 5 Paul Flechsig Institute for Brain Research, University of Leipzig, Leipzig, Germany, and Leibniz Institute for Age Research, Jena, Germany; 6 Institute for Neuropathology, Friedrich-Alexander-Universität Erlangen-Nürnberg, Erlangen, Germany; 7 Department of Neurology, Friedrich-Alexander-Universität Erlangen-Nürnberg, Erlangen, Germany; 8 Fritz Lipmann Institute, Molecular Motors Lab, Jena, Germany; University of Wurzburg, Germany

## Abstract

Swiprosin-1/EFhd2 (EFhd2) is a cytoskeletal Ca^2+^ sensor protein strongly expressed in the brain. It has been shown to interact with mutant tau, which can promote neurodegeneration, but nothing is known about the physiological function of EFhd2 in the nervous system. To elucidate this question, we analyzed EFhd2^−/−^/*lacZ* reporter mice and showed that *lacZ* was strongly expressed in the cortex, the dentate gyrus, the CA1 and CA2 regions of the hippocampus, the thalamus, and the olfactory bulb. Immunohistochemistry and western blotting confirmed this pattern and revealed expression of EFhd2 during neuronal maturation. In cortical neurons, EFhd2 was detected in neurites marked by MAP2 and co-localized with pre- and post-synaptic markers. Approximately one third of EFhd2 associated with a biochemically isolated synaptosome preparation. There, EFhd2 was mostly confined to the cytosolic and plasma membrane fractions. Both synaptic endocytosis and exocytosis in primary hippocampal EFhd2^−/−^ neurons were unaltered but transport of synaptophysin-GFP containing vesicles was enhanced in EFhd2^−/−^ primary hippocampal neurons, and notably, EFhd2 inhibited kinesin mediated microtubule gliding. Therefore, we found that EFhd2 is a neuronal protein that interferes with kinesin-mediated transport.

## Introduction

Ca^2+^ ions are crucial second messengers in synaptic transmission and cytoskeleton function. Ca^2+^ binding proteins of different classes play important roles in the brain [1]. Swiprosin-1/EFhd2 (EFhd2) is a proposed Ca^2+^ sensor protein expressed abundantly in the brain [2], [3], and was shown to interact with tauP301L [4]. EFhd2 consists of a N-terminal region of low complexity with an alanine stretch, a functional SH3 binding motif [5], two functional EF hands [2], [6] and a C-terminal coiled-coil domain [7]. The coiled-coil domain of recombinant EFhd2 is responsible for self-oligomerization in a Ca^2+^-dependent manner [8] and for the interaction with mutant tau in brain extracts of JNPL3 mice *in vitro* [9]. How EFhd2 may be linked with neurodegenerative diseases is not known, due to the physiological function of EFhd2 being poorly understood. Though it was previously reported that EFhd2 is part of conserved functional cytoskeletal and Ca^2+^ feedback loops in B cells and other systems [2], [10], which within neurons would point towards functions in neurite maintenance and/or transport.

Anterograde and retrograde axonal transport along microtubules (MTs) of most cargos is mediated by the motor proteins kinesin and dynein, respectively [11]. Microtubule associated proteins (MAP) assist these processes by stabilizing MTs. The MAP tau directly binds and thus stabilizes MT in axons and microtubule associated protein 2 (MAP2) stabilizes MT in dendrites [11], [12]. Defects in synaptic and transport proteins are involved in neurodegenerative diseases by interfering with axonal transport and neural circuit function (reviewed in [13], [11]). Axonal branching during physiological axonal regeneration requires local destabilization of the MT cytoskeleton [12]. This process requires detachment of tau from MTs, which can be mediated by the phosphorylation of tau at many residues [12]. Therefore, controlled tau phosphorylation is a critical physiological process and may be linked with EFhd2 [4].

We therefore tested the hypothesis that EFhd2 controls cytoskeletal functions in neurons using EFhd2 knock-out/*lacZ* knock-in mice. We found that EFhd2 was strongly expressed in the cortex, hippocampus, thalamus and the olfactory bulb. We revealed that EFhd2 is has a negative impact on transport of synaptophysin-GFP containing vesicles in hippocampal neurons. Specifically, EFhd2 inhibited kinesin mediated microtubule gliding. Taken together, we propose that EFhd2 is a neuronal protein that interferes with kinesin activity.

## Materials and Methods

### Chemicals

All chemicals were purchased from Sigma-Aldrich (Deisenhofen; Germany) Merck (Darmstadt; Germany) or Roth (Karlsruhe; Germany) unless stated otherwise. Cell culture medium and supplements were obtained from Invitrogen Life Technologies (Heidelberg, Germany).

### Mice

Mice were maintained and sacrificed in accordance with the European Communities Council Directive of 24^th^ Nov. 1996 and were approved by the local governmental administration for animal health (Permit number: 54.2531.31-28/08, government of the administrative region of Mittelfranken; Genehmigungen zur Haltung genetisch veränderter Tiere, permit number 820-8791.2.41, government of the administrative region of Unterfranken). All efforts were made to minimize suffering. Wild type (WT) C57BL/6 mice at embryonic (E16 and E18) and adult ages (P150) were used. We have generated mice deficient for EFhd2 on a C57Bl/6 background using stem cells from the trans-NIH Knock-Out Mouse Project (KOMP, https://www.komp.org/) from Velocigen Regeneron Pharmaceuticals, and described elsewhere [14].

### Immunohistochemistry and β-Galactosidase staining

For immunohistochemistry, brains from adult mice (P150) were removed, fixed in 4% para-formaldehyed (PFA) in phosphate-buffered saline (PBS) and embedded in paraffine. Tissue sections (8 µm) were deparaffinized and antigen was retrieved [15]. Briefly, sections were heated for 30 min in a Benchmark-Stainer (Roche) in 10 mM Tris, 1 mM EDTA, 0.05% Tween 20 at 95°C. Sections were then blocked in 1% bovine serum albumin (BSA) in PBS and incubated with undiluted hybridoma supernatant containing EFhd2 mouse antibody (mAb) [16] and anti-mouse Fcγ specific antibody coupled to horseradish peroxidase (HRP, Jackson). To visualize β-Galactosidase activity, brains were fixed in 0.2% Glutaraldehyde, 2 mM MgCl_2_, 5 mM EDTA in PBS for 1 h at room temperature (RT), washed twice for 30 min in 2 mM MgCl_2_, 5 mM EDTA in PBS, and incubated in staining solution (2 mM X-Gal, 5 mM potassium hexacyanido ferrate (II), 5 mM potassium hexacyanido ferrate (III), 2 mM MgCl_2_, 5 mM EDTA in PBS) for 2 h at 37°C. Brains were washed 3 times for 5 min in PBS, fixed over-night (ON) in 4% PFA, and washed 3 times for 15 min in PBS. Then, the brains were incubated for 1 h each in 50%/70%/80% ethanol, and analyzed as whole mount or embedded in 4% agarose and coronally sectioned (100 µm) using a vibratome (Leica RM2055). Sections were examined with an inverted microscope (Zeiss Apotome 2) using Zeiss Axiovision 4.8 software (Carl Zeiss).

### Primary Cortical Neurons

Cortices from mouse embryos at E16 were dissected, chopped into 200–500 µm pieces and further mechanically dissociated with siliconated glass pipettes. Neurons were seeded at a density of 1×10^5^ cells/cm^2^ and cultured in Neurobasal culture media (Invitrogen) supplemented with L-Glutamine, 2% B27 and Penicillin-Streptomycin in 5% CO2, 95% humidity at 37°C. Neurons were transfected with constructs encoding dTomato and EFhd2Myc [3] using Lipofectamine 2000 (Invitrogen) following the manufacturer's instructions. For immunostainings, neurons were fixed in 4% PFA. Non-specific binding sites were blocked with immunofluorescence buffer (IFB; PBS supplemented with 0.1% Triton x-100 and 5% normal donkey serum) for 1 h at RT. Neurons were stained ON with anti-EFhd2 mAb (1∶200). Synapses were labelled with goat anti-synapsin 1a/b antibody (1∶1000; Santa Cruz), rabbit and goat anti-PSD95 (postsynaptic density protein 95) antibodies (1∶200 each; Abcam), rabbit anti-VAMP2 (vesicle-associated membrane protein 2; 1∶2000; Synapse Systems). Neurites were stained with goat anti-tau (microtubule-associated protein tau; 1∶250; Santa Cruz) and rabbit anti-MAP2 (microtubule-associated protein 2; 1∶200; Abcam). Next, the samples were rinsed several times with PBS supplemented with 0.1% Triton x-100, and incubated with suitable fluorescently labelled secondary antibodies (Invitrogen) diluted in IFB. After mounting, cells were either analyzed by confocal microscopy (Zeiss LSM780, digital resolution 1024×1024) or with a Zeiss Apotome 2 microscope. The co-localization of EFhd2 with Tau and MAP2 was assessed using ImageJ and Volocity (Perkin-Elmer) as previously described in detail [17], [18] and is expressed as Pearson's co-localization co-efficient. Co-localization analysis of EFhd2/synapsin and EFhd2/PSD95 was performed with deconvoluted images derived from an Olympus Deltavision microscope (60×; NA 1.42), using the JACOP plugin in ImageJ (sbweb.nih.gov/ij/), which provides the Pearson's co-localization co-efficient.

### Synaptosome preparation by centrifugation

Synaptosomes were prepared as previously published [18], [19]. Briefly, forebrains from three to four adult mice were dissected out and kept in a cold glass-Teflon homogenizer together with 30 ml of pre-chilled 4 mM Hepes-NaOH; pH 7.3 supplemented with 0.32 M sucrose. Tissue homogenization was performed by 10 up-down strokes at 900 rpm and the tissue homogenate was centrifuged at 800×*g* for 10 min at 4°C. The resulting supernatant (S1) was centrifuged at 10,000×*g* for 15 min at 4°C, resuspended and centrifuged again for 20 min 100000×*g* to obtain pellet 2 (P2) representing the crude synaptosome fraction (STS), and supernatant (S2). Synaptosomes were broken by osmotic shock, and the synaptosomal plasmatic membranes (P3; PM) separated by centrifugation at 25,000×*g* for 20 min at 4°C. The resulting supernatant (S3) was further centrifugated at 30,000×*g* ON at 4°C in order to separate the cytosolic fraction (S4; Cyt) and the synaptic vesicles fraction (P4; SV). Synaptosomal fractions were further analyzed by western blot.

### Life cell imaging of hippocampal neurons

Hippocampal neuronal cultures were prepared from one to three days old EFhd2^+/+^ and EFhd2^−/−^ mice [14]. Newborn mice were killed by decapitation. Hippocampi were removed from the brain and transferred into ice-cold Hank's salt solution, and the dentate gyrus was removed. After digestion with trypsin (5 mg/ml), cells were triturated mechanically and plated in MEM medium, supplemented with 10% fetal calf serum and 2% B27 Supplement (all Invitrogen). Neurons were transfected with synaptophysin-EGFP under control of a synapsin promoter on day 3 *in vitro* (DIV) with a modified calcium phosphate method as described [20], [21]. Experiments were performed on day DIV 23.

Imaging was performed as described previously (Welzel et al., 2009; Welzel et al., 2011). Experiments were conducted at room temperature on a Nikon TI-Eclipse inverted microscope equipped with a 60×,1.2 NA water immersion objective and Perfect Focus System. Fluorescent dyes were excited by a Nikon Intensilight C-HGFI through an excitation filter centered at 482 nm using a dichroic longpass mirror (cut-off wavelength 500 nm). The emitted light passed an emission band-pass filter ranging from 500–550 nm (Semrock, Rochester) and was projected onto a cooled EM-CCD camera (iXon DU-885, Andor). Cover slips were placed into a perfusion chamber (volume = 500 µl) containing extracellular medium containing (in mM): 144 NaCl, 2.5 KCl, 2.5 CaCl_2_, 2.5 MgCl_2_, 10 Glucose, 10 Hepes, pH 7.5. Synaptic boutons were stimulated by electric field stimulation (platinum electrodes, 10 mm spacing, 1 ms pulses of 50 mA and alternating polarity); 10 µM 6-cyano-7-nitroquinoxaline-2,3-dione (CNQX, Tocris Bioscience) and 50 µM D-amino-5-phosphonovaleric acid (D,L-AP5, Tocris Bioscience) were added to prevent recurrent activity. Images were recorded with 200 ms exposure time at 1 Hz frame rate. In all experiments resulting image stacks were converted into ICS file format. Kymographs were generated using the dropdown menu “kymograph” in MetaMorph 7.5.1. (MolecularDevices). Only straight axonal segments were selected for analysis to avoid velocity artifacts originating from factors such as strong axon covings. Kymographs were analyzed using the Hough transform as described previously (Welzel et al., 2011).

### Protein purification

GST fusion proteins encoded in pGEX2T (GE healthcare) were induced and purified as described previously [2], [16] and dialyzed into appropriate buffers. A truncated form comprising 560 amino acids from the N-terminus and containing the motor domain, neck and part of the stalk of human neuron-specific kinesin-1 (KIF5A_560_) was expressed in *E.coli* as fusion protein with a C-terminal chitin-binding intein tag and purified by binding to chitin beads followed by removal of the intein-tag by 50 mM DTT-induced specific self-cleavage [22]. Kinesin was concentrated using Amicon ultra filter units (Merk Millipore) and stored in motility buffer (50 mM imidazole, 0.5 mM MgCl_2_, 0.5 mM EGTA, 0.5 mM DTT, pH 6.8) supplemented with 150 mM NaCl and 1 M glycerol at −80°C. Tubulin was purified from porcine brain homogenates by two cycles of temperature-dependent disassembly at 0°C and reassembly at 37°C. Co-purified MAPs were removed by phosphocellulose ion exchange chromatography [23]. Stabilized MTs were formed by taxol-promoted self-assembly at 37°C from 2 mg/ml pure tubulin in the MT assembly buffer (20 mM PIPES, 80 mM NaCl, 1 mM EGTA, 0.5 mM MgCl_2_, pH 6.8) supplemented with 0.2 mM GTP and 20 µM taxol. MTs were usually prepared at the day of usage at least 30 min prior to an experiment. The purity of kinesin was 75–80%, the purities of GST, GST-EFhd2 and tubulin were all 95–98% as determined by SDS-PAGE.

### *In vitro* gliding assay

Gliding assays were performed as described previously [24], [25] MTs were assembled and stabilized as described above. All gliding assays were performed with pure KIF5A_560_ in motility buffer (26.5 mM Imidazol, 9.4 mM PIPES, 0.735 mM EGTA, 0.265 mM DTT, 137.6 mM NaCl, 1.5 mM MgCl_2_, 0.5 mM ATP, and 0.004 mM GTP) supplemented with 20 µM taxol. The gliding assay mixture containing KIF5A_560_ (0.4 mg/ml), stabilized MTs (40 µg/ml), and GST or GST-EFhd2 at indicated concentrations was incubated 5 min at room temperature prior transferring it onto casein-coated glass slides. The sample was covered by a cover slip. MT gliding was visualized by video-enhanced differential interference contrast microscopy; gliding velocities were calculated from at least 10 MTs in each sample using the Argus-20 software (Hamamatsu Photonics). 100% correspond to the gliding velocity of 1.3 um/sec. All analyses were performed using a Zeiss Axiophot microscope (Carl Zeiss) equipped with a Plan-Neofluor 63×/1.25 oil immersion objective (Carl Zeiss), a Chalnicon video camera type C2400-0.1, and the image processing system Argus-20 (both Hamamatsu Photonics), which enables background subtraction, and electronic contrast enhancement. Microtubule movement was documented on a digital hard-disk recorder (JVC SR-DVM70EU, JVC).

### Western blotting

Brains were extracted in cold homogenization buffer (50 mM Tris, pH 8.0; 2 mM EDTA, 140 mM NaCl) supplemented with 1 mM dithiothreitol (DTT) as well as protease and phosphatase inhibitors. After homogenization, samples were centrifuged first at 800×*g* for 10 min. Supernatants (S1) were incubated with 1% of Triton x-100 for 30 min on ice and centrifuged at 11,000×*g* for 10 min at 4°C. Neurons were directly lysed in homogenization buffer containing 1% Triton X-100. 5 to 40 µg of total protein were loaded were separated by SDS-PAGE and blotted onto nitrocellulose membranes. Primary antibodies were incubated ON at 4°C in Tris-buffered saline and 0.1% Tween20 (TBST) supplemented with 3% BSA (mouse anti-SNAP-25 (clone SMI-81; 1∶1000; Synaptic Systems), mouse anti-synaptophysin (clone p38; 1∶1000; Sigma-Aldrich); mouse anti-β3-tubulin (TUJ 1∶2000; Covance); goat anti EFhd2 (1∶500; Everest Biotech), rabbit anti EFhd2 ([26], 1 µg/ml); goat anti Tau (1∶500; Santa Cruz) were employed to determinate synaptosomal sub-fractions. After several washes with TBST, blots were incubated with corresponding secondary antibodies conjugated to horseradish peroxidase and developed by chemical luminescence techniques.

### Statistical Analysis

Data are expressed as mean ± standard error of the mean (SEM). Statistical analysis was done with the Student's t-test for unpaired variables (two-tailed) when data passed the Kolmogorov-Smirnov Test (with Dallal-Wilkinson-Lilliefor P value) or otherwise, as indicated in the figure legends or the main text. Statistics were calculated using GraphPad Prism software. *P*-values≤0.05 were considered significant (*). ** and ***: p<0.001 and 0.0002, respectively.

## Results

### EFhd2 is expressed in the murine cortex and hippocampus

**Figure 1:**
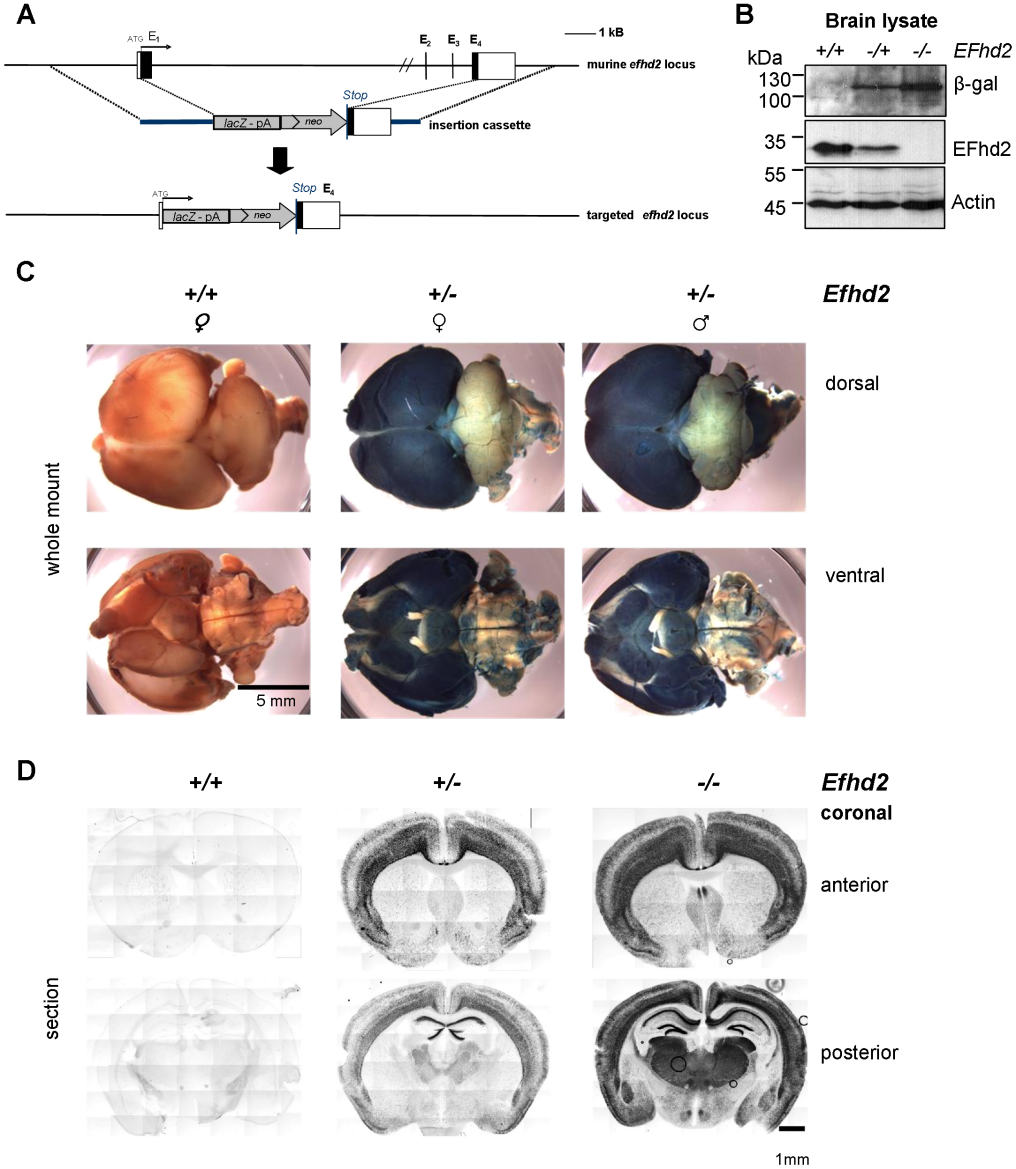
EFhd2 expression in adult mouse brain determined by *lacZ* reporter gene expression. (*A*) Replacement of the murine *efhd2* locus by a *lacZ* reporter gene cassette. E1-4: Exons. Black: coding regions in exons. (*B*) Lysates of brains of wild-type (+/+), heterozygous (+/−) or EFhd2-deficient mice (−/−) were subjected to western blot analysis with antibodies indicated on the right. (*C*) Whole mount *lacZ* reporter gene staining of brains of adult mice. (*D*) Coronal sections of anterior and posterior parts of whole mount stained brains from adult EFhd2^+/+^, EFhd2^+/−^ andEFhd2^−/−^ mice.

To determine the expression of EFhd2 in the adult brain we utilized an EFhd2^−/−^ knockout/*lacZ* knock-in mouse strain generated in our laboratory [14]. In heterozygous animals (EFhd2^+/−^), one *efhd2* allele is replaced by a *lacZ* reporter gene (Fig. 1A). The *lacZ* reporter gene downstream of the *efhd2* promoter, resulting in β-galactosidase expression, indicates *efhd2* mRNA expression, which we have shown to be strong compared to other tissues [3]. Hence, EFhd2^−/+^ and EFhd2^−/−^ mice express β-galactosidase in the adult brain, while protein levels of EFhd2 are reduced correspondingly (Fig. 1B). Whole mount staining revealed strong β-galactosidase activity in the forebrain (Fig. 1C). To determine *efhd2* expression in more detail, we sectioned whole mount stained adult mouse brains coronally (Fig. 1D). Prominent dose-dependent β-galactosidase activity indicative of *efhd2* promoter activity was detected in the deeper layers of the cortex (IV, V, and VI), as well as the dentate gyrus, the CA1 and CA2 areas of the hippocampus. β-galactosidase activity was also observed in the thalamus and the olfactory bulb (Fig. 1D).

### EFhd2 protein is expressed in embryonic brain tissue and primary cortical neurons

**Figure 2:**
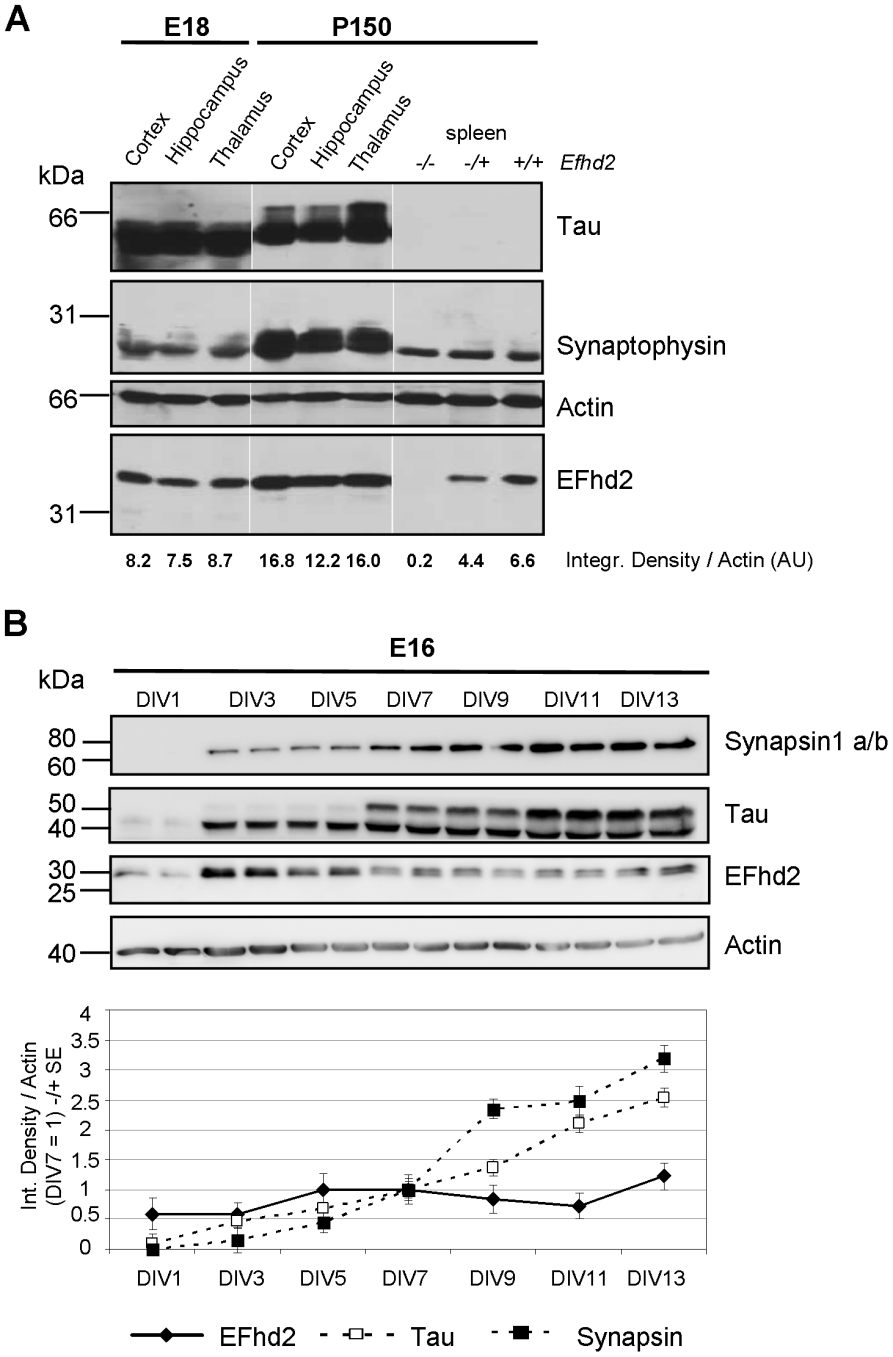
EFhd2 protein expression in embryonic and adult brain regions as well as in cortical neurons. (*A*) Embryonic (E18) and adult brains (P150) of wildtype mice were dissected into indicated regions. These regions as well as spleens from adult EFhd2^−/−^, ^−/+^ and ^+/+^ mice were lysed and lysates were subjected to 10% SDS-PAGE, followed by western blotting with polyclonal antibodies indicated on the right. Molecular mass standards are indicated on the left (kDa). Optical densities of EFhd2 bands were normalized to actin bands. Representative of three experiments. (*B*) Cultures of E16 cortical neurons were cultured for the indicated days *in vitro* (DIV) and lysed. Each time point is represented by two lanes showing two independent cultures. Lysates were subjected to 10% SDS-PAGE, followed by western blotting with antibodies indicated on the right. Molecular mass standards are indicated on the left (kDa). Optical densities of synapsin1a/b, tau and EFhd2 bands were normalized to actin (n = 8 from 4 experiments; one representative experiment is shown). Data are represented as mean −/+ SEM.

Next, we showed the presence of the EFhd2 protein in the cortex, hippocampus, and thalamus of wild-type mouse embryos (E18) and adult tissue (P150) by western blot analysis (Fig. 2A). Importantly, we noted that EFhd2 was expressed in all forebrain areas at embryonic and adult stages (Fig. 2A), the latter of which confirmed previous findings [4], but embryonic expression has not been revealed before. As a control, we included non-neuronal tissue (spleen) of EFhd2^+/+^, ^−/+^ and ^−/−^ mice, confirming the predominant expression of EFhd2 protein in the adult brain compared to other organs [3]. Next, we examined the expression of EFhd2 in cortical neurons, where we observed a moderate up-regulation of EFhd2 between DIV1 and DIV13 (compare EFhd2 expression relative to Actin), concomitant with steady up-regulation of tau and the pre-synaptic marker synapsin (Fig. 2B). Hence, EFhd2 is expressed in embryonic and adult brain as well as in primary cortical neurons. The EFhd2 protein was up-regulated roughly two-fold during neuronal maturation *in vitro* and in parallel with tau. In complete accordance, EFhd2 protein was increased roughly two-fold in adult versus embryonic brain tissue.

**Figure 3:**
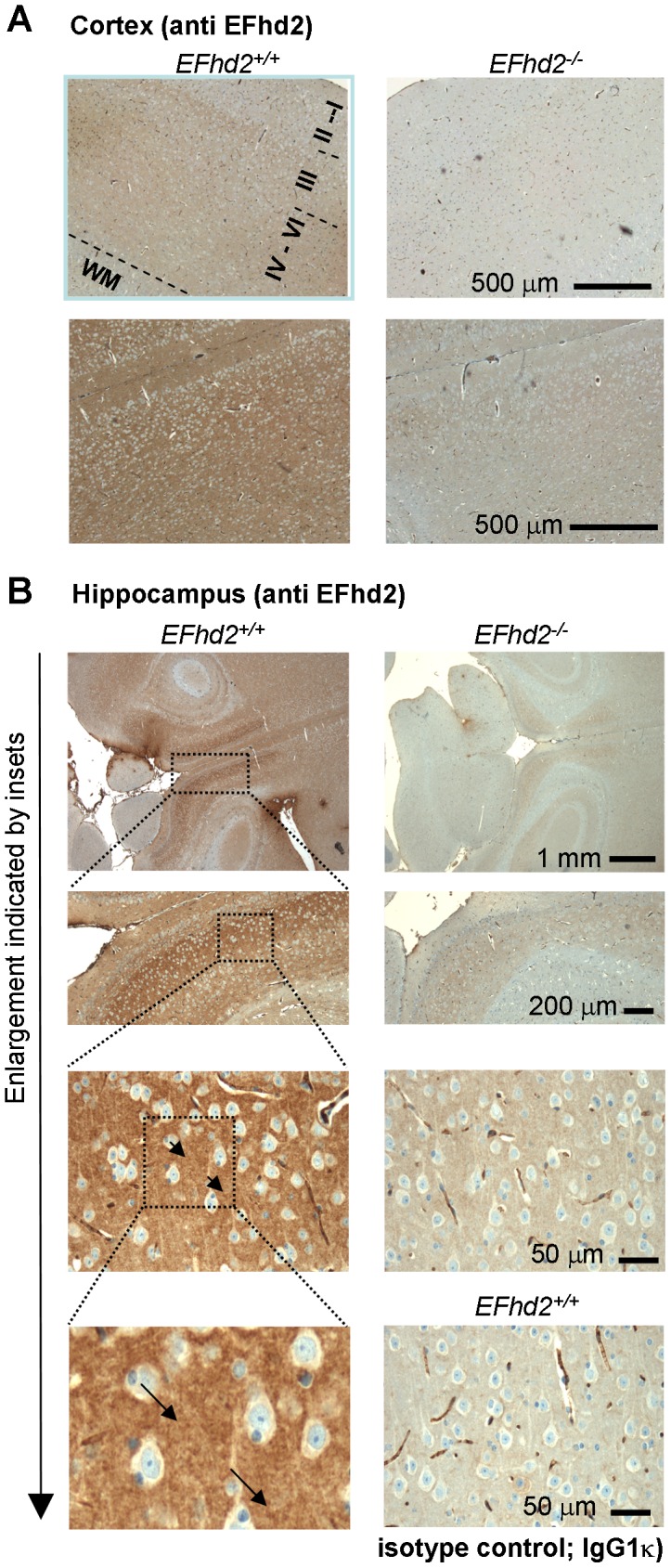
Specific detection of EFhd2 protein in murine brain sections. Brains of EFhd2^+/+^ and EFhd2^−/−^ mice were fixed, sectioned (horizontal) and stained with anti EFhd2 monoclonal antibody A4.18.18 in the cortex (*A*) or hippocampus (*B*), or in the hippocampus with an isotype matched control antibody (IgG1k, *C*). Antibodies were detected by secondary anti mouse antibody coupled to horseradish peroxidase and diaminobenzidine staining. Nuclei were counterstained with Alcian Blue. The pyramidal layers (layers III–V) and the multiform layer (layer VI) of the cortex, the molecular layer (I), the external granular layer (II) and the white matter are indicated. Representative of at least 10 mice of each genotype. Similar results were obtained with 2 additional different anti EFhd2 mAb (E7.20.23; A4.15.48) [16].

Immunohistochemistry confirmed the expression of EFhd2 in the pyramidal layers (layers III–V) and the multiform layer (layer VI) of the cortex (Fig. 3A). Conversely, EFhd2 was never detected in the molecular layer (layer I), the external granular layer (layer II) and the white matter (Fig. 3A). The EFhd2 signal was also specific in the granular and pyramidal cell laminas of the hippocampus (Fig. 3B). EFhd2 was associated with cell bodies but apparently not nuclear. A dot-like neuropil staining (see arrowheads in Fig. 3B) of EFhd2 was observed in the hippocampus of EFhd2^+/+^ mice but not of EFhd2^−/−^ mice using anti-EFhd2 mAb A4.18.18 [16]. Similar results were obtained with the anti-EFhd2 mAb A4.15.18 [16] (data not shown). An isotype matched control antibody (IgG1κ) did not reveal a specific staining. The intercellular, dot-like expression of EFhd2 indicated a staining of neurites or synapses, and we next tested these possibilities in cultures of primary neurons.

### EFhd2 is expressed in neurites marked by tau and MAP2 in murine cortical neurons and is found in proximity to pre- and post-synaptic markers

**Figure 4:**
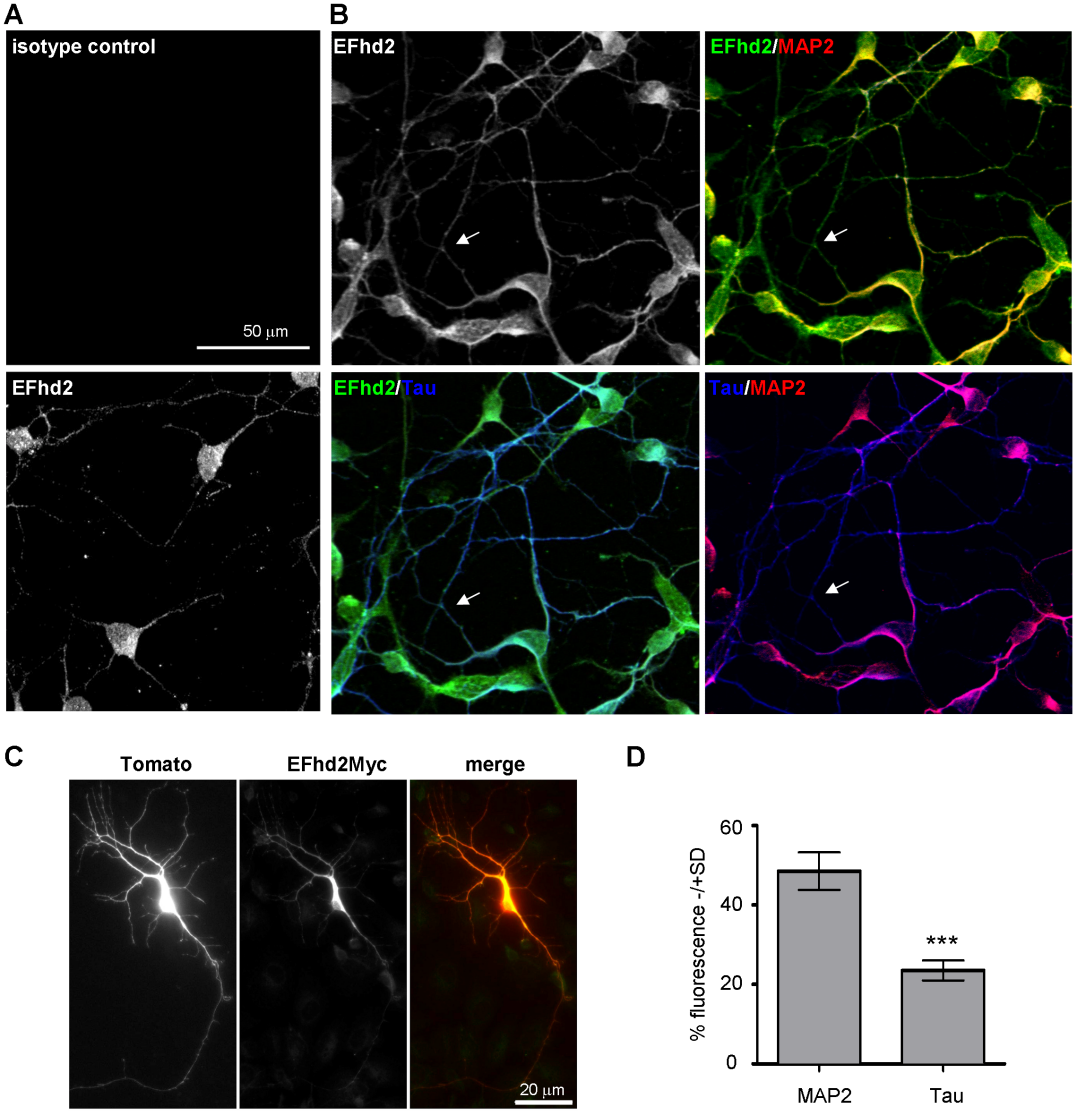
Co-localization of EFhd2 with neurites marked by tau and MAP2 in murine cortical neurons. (*A*) Murine cortical neurons (DIV7) were stained with the primary antibodies anti EFhd2 or an isotype matched control antibody (IgG1k) and analyzed by confocal microcsopy. (*B*) Murine cortical neurons were stained with the primary antibodies anti EFhd2, tau and MAP2. Single images were taken by confocal microscopy and merged (right panel). (*C*) Murine cortical neurons were double-transfected with constructs encoding dTomato (red) and Myc-tagged EFhd2 (EFhd2Myc; green) and images were taken with an Apoptome. (*D*) Quantification of the sub-neuronal distribution of EFhd2; data are represented as mean ± SEM (*** *P*<0.001; two-tailed student's t-test).

**Figure 5:**
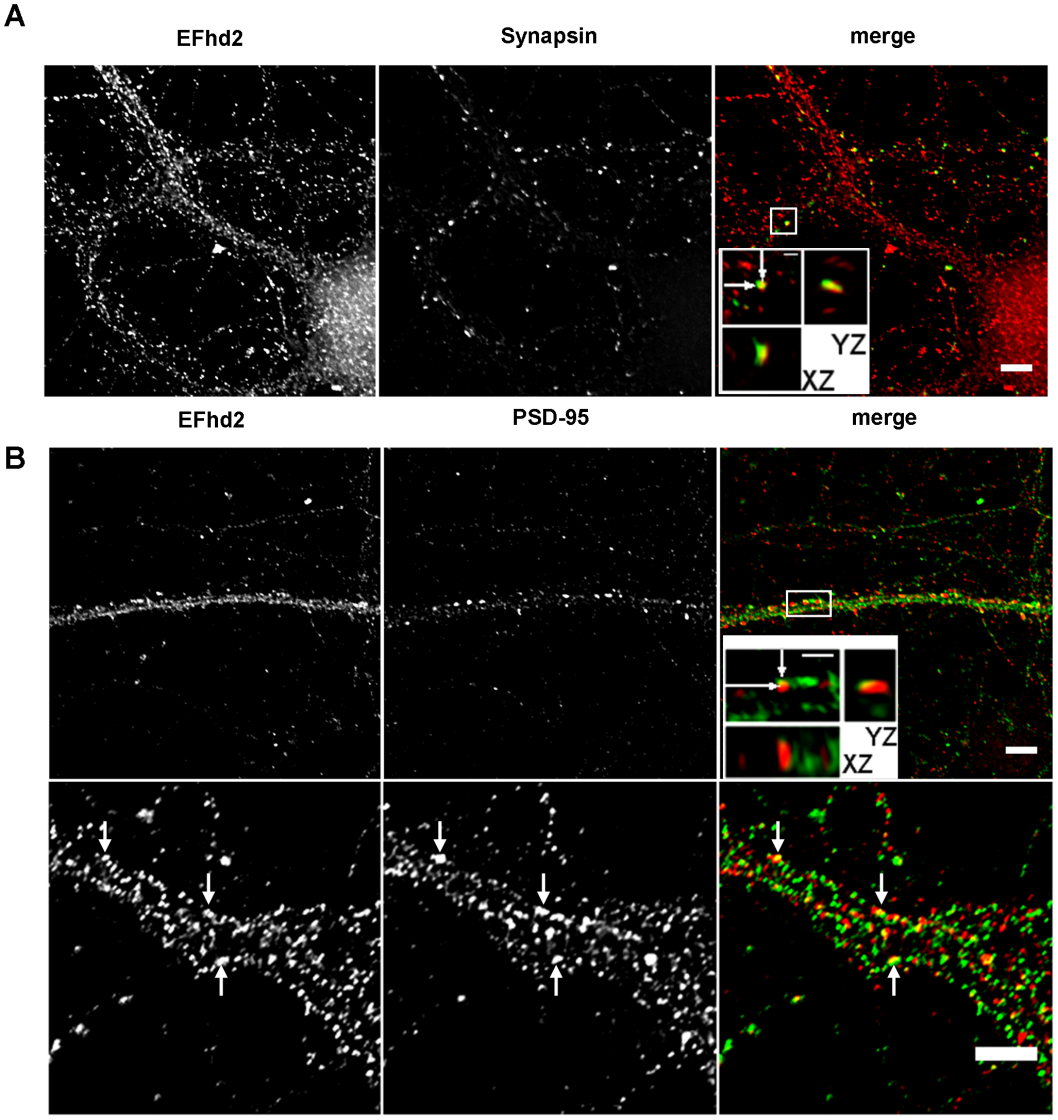
Co-localization of EFhd2 with Synapsin and PSD95 revealed deconvolution microscopy. (*A* and *B*) DIV11 and DIV13 cortical neurons were fixed and stained with anti EFhd2 mAb (green), anti-synapsin 1a/b (red) (*A*) and rabbit anti-PSD95 (red) (*B*) antibodies. Mounted cells were analyzed by deconvolution microscopy. Selected areas (insets) were enhanced and visualized in xy and yz dimensions. Images represent 0.2 µm slices of from a deconvolved image stack. Scale bars represent 5 µm (1 µm in the insets).

We therefore analyzed localization of EFhd2 in primary cortical neurons by confocal microscopy. The anti-EFhd2 mAb specifically stained the somatodendritic region and neurites in a punctuate fashion (Fig. 4A). This staining resembled the EFhd2 staining pattern observed *in situ* (Fig. 3B). To determine whether EFhd2 is associated with axons, which was anticipated due to the previously published interaction with tau [4], or with dendrites, we performed co-staining of EFhd2 with tau and MAP2 [27] (Fig. 4B). This experiment revealed a distinct staining of MAP2 in neurites and the somatodendritic cytoplasm. There were also neurites that were only positive for tau (see arrows in Fig. 4B), as the establishment of neuronal polarity, i.e. axon-dendrite specification, has taken place under the culture conditions we applied [27], [28]. Interestingly, EFhd2 revealed a clear co-localization with MAP2 when compared to the isotype control (Fig. 4B). However, EFhd2 was also present in neurites marked with tau. Quantification of the fluorescence signals showed that EFhd2 was associated with neurites marked by tau to about 30%, but mostly with neurites marked by MAP2, to about 50% (Fig. 4B; summary in Fig. 4D). The staining results obtained with the anti-EFhd2 mAb (dominant staining of MAP2 marked neurites) were confirmed by Myc-staining in neurons transfected with a Myc-tagged EFhd2 protein [3] (Fig. 4C). We selectively chose neurons that moderately over-expressed EFhd2Myc. In summary, EFhd2 is not only associated with tau marked neurites, as might have been inferred from the interaction with mutant tau [4], but also with neurites marked by MAP2 as well as the cytoplasm of primary neurons. To further elaborate this result, we investigated whether EFhd2 might be associated with the pre- and postsynaptic markers synapsin 1 a/b and PSD95 by deconvolution microscopy. Indeed, there was a partial co-localization of EFhd2 with the pre-synaptic marker synapsin 1a/b [29] (Fig. 5A). Quantification revealed that the fluorescence signals of EFhd2 and synapsin 1a/b overlapped with a Pearson's co-localization coefficient of 0.49. Vice versa, the fluorescence signals of synapsin 1a/b overlapped with EFhd2 signals with a Pearson's co-localization coefficient of 0.55 (Fig. 5). In addition, EFhd2 was found in close association with PSD-95 (Fig. 5B), confirming its dendritic localization, but did not directly co-localize (Pearson's co-localization coefficients EFhd2/PSD-95: 0.31, PSD-95/EFhd2: 0.14). Hence, using two different marker systems (MAP2/Tau and Synapsin1/PSD95) we reveal that EFhd2 is localized in neurites. The punctuate staining pattern along neurites, and the staining pattern *in situ* (Fig. 3B) suggested that EFhd2 colocalizes with transport vesicles; or with synaptic structures; or both. Hence, EFhd2 could be associated with transport vesicles delivering synaptic proteins but also be a synaptic protein. These possibilities are not mutually exclusive.

### EFhd2 is associated with the cytosolic and plasma membrane fractions of biochemically isolated synaptosomes

**Figure 6:**
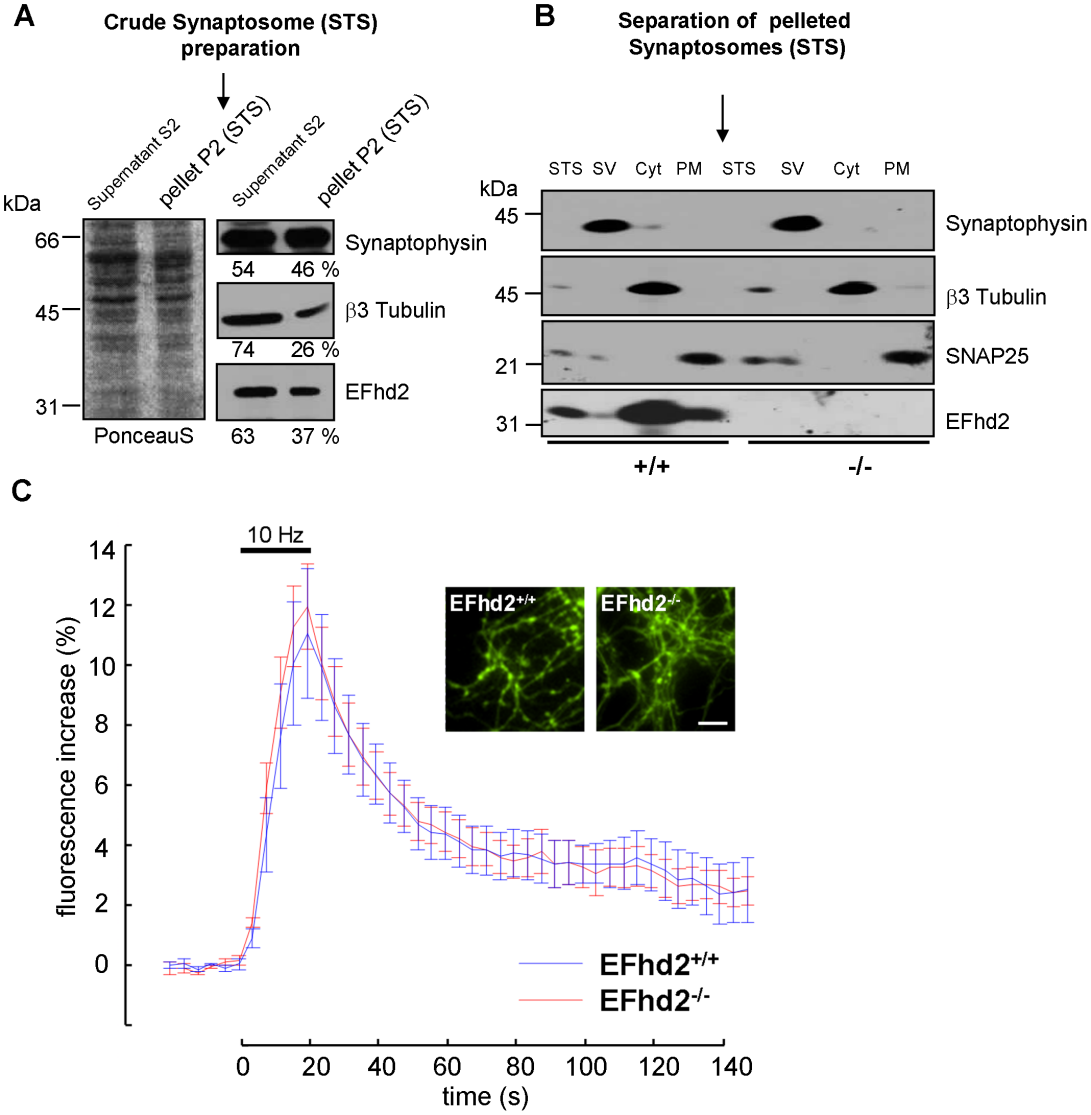
Synaptic localization of EFhd2 is not required for synapse function in primary hippocampal neurons. (*A*) Crude synaptosomes were purified from pooled brains and centrifuged. Equal amounts of the supernatant (S2) and the crude synaptosome fraction (STS; P2) were subjected to 10% SDS-PAGE, followed by Western Blotting, Ponceau S staining of the membrane and incubation with antibodies as indicated on the right. The relative distribution of a cytosolic protein (β3-tubulin), a synaptic marker (synaptophysin) and EFhd2 in synaptosomes was calculated by OD measurement. (*B*) Equal amounts of protein were obtained from each of the separated synaptosomal (S) fractions (Cyt: cytosolic fraction, PM: plasma membrane, SV: synaptic vesicles) and subjected to 10% SDS-PAGE, followed by western blotting with antibodies indicated on the right. β3-tubulin is a marker protein for the cytosolic fraction (Cyt), synaptophysin is a marker protein for synaptic vesicle (SV) fractions and SNAP25 is a marker for plasma membrane (PM) and synaptic vesicle fractions. Molecular mass standards are indicated on the left (kDa). Representative of two independent experiments performed with each three EFhd2^+/+^ and three EFhd2^−/−^ mice. (*C*) Normalized fluorescence intensity profiles of synapto-phluorin transfected primary neurons of EFhd2^+/+^ and EFhd2^−/−^ mice. Neurons were stimulated with 200 pulses at 10 Hz. Insets show exemplary fluorescence images. Error bars indicate SD. Scale bar, 10 µm. n = 6 transfections.

To test the possibility that EFhd2 is associated with synapses, we analyzed synaptosomes prepared by differential centrifugation [18], [19] from brains of adult EFhd2^+/+^ and EFhd2^−/−^ mice. First, we assessed to which extent EFhd2 could associate with this biochemically isolated brain fraction. To this end we analyzed the amount of EFhd2 protein present in the pelleted fraction (P2 – STS [synaptosomes]) that was further used to obtain the different synaptosome fractions and the fraction S2 (not used for further analysis). Whereas the cytosolic protein β3-tubulin was only to 26% in the P2 fraction, the synaptic marker synaptophysin was present much more, and EFhd2 was present to 37%, indicating that a substantial part of EFhd2 can associate with biochemically prepared synaptosomes (Fig. 6A). Next, we assessed where in synaptosomes EFhd2 was located. Therefore, the fraction P2 synaptosomes were separated into cytosolic (Cyt), synaptic vesicle (SV) and plasma membrane (PM) fractions. Using protein markers for specific fractions, namely β3-tubulin - cytosolic fraction, synaptophysin - synaptic vesicle fraction and SNAP25 - plasma membrane [29], we revealed that EFhd2 is present mostly in the cytosolic and synaptic plasma membrane compartments. The abundance of the synaptic proteins synaptophysin and SNAP25 was unchanged between EFhd2^−/−^ mice and controls (Fig. 6B). Taken together, these data revealed that a fraction of EFhd2 is localized in biochemically isolated synapses [19]. We did not control these fractions by electron microscopy as in the original publication. Importantly, however, synapsin, s a synaptic marker, is also found in these preparations [19] and we found that EFhd2 co-localizes with synapsin (Fig. 4A) in matured primary neuronal cultures [27]. Thus, our imaging and our biochemical approach support the notion that EFhd2 associates with vesicular structures that might be part of - or be delivered to - synapses. Ultrastructural analyses *in vitro* and *in situ* will be necessary to address the question of specific synaptic abundance or accumulation of EFhd2 in the future.

### EFhd2 does not impact endocytosis and exocytosis

To test the possibility that lack of the low affinity Ca^2+^ binding protein EFhd2 [2] alters synaptic functions, we transfected primary neurons of EFhd2^+/+^ and EFhd2^−/−^ mice with synapto-phluorin [30] and monitored synaptic exo- and endocytosis following an electrical stimulus with 200 pulses at 10 Hz (Fig. 6C). This experiment demonstrated that neither the amount of exocytosed synaptic vesicles (ΔF/F_0,*τ*+/+_ = 11.08±5.25%, ΔF/F_0,*τ*−/−_ = 11.20±3.48%; two-sample *t*-test: *p* = 0.73) nor the endocytosis and related kinetics (*τ*_+/+_ = 27.17±4.56 seconds, *τ*_−/−_ = 28.01±2.45 seconds; two-sample *t*-test: *p* = 0.87) were affected by the presence of EFhd2.

### EFhd2 slows down axonal transport and inhibits kinesin activity *in vitro*

**Figure 7:**
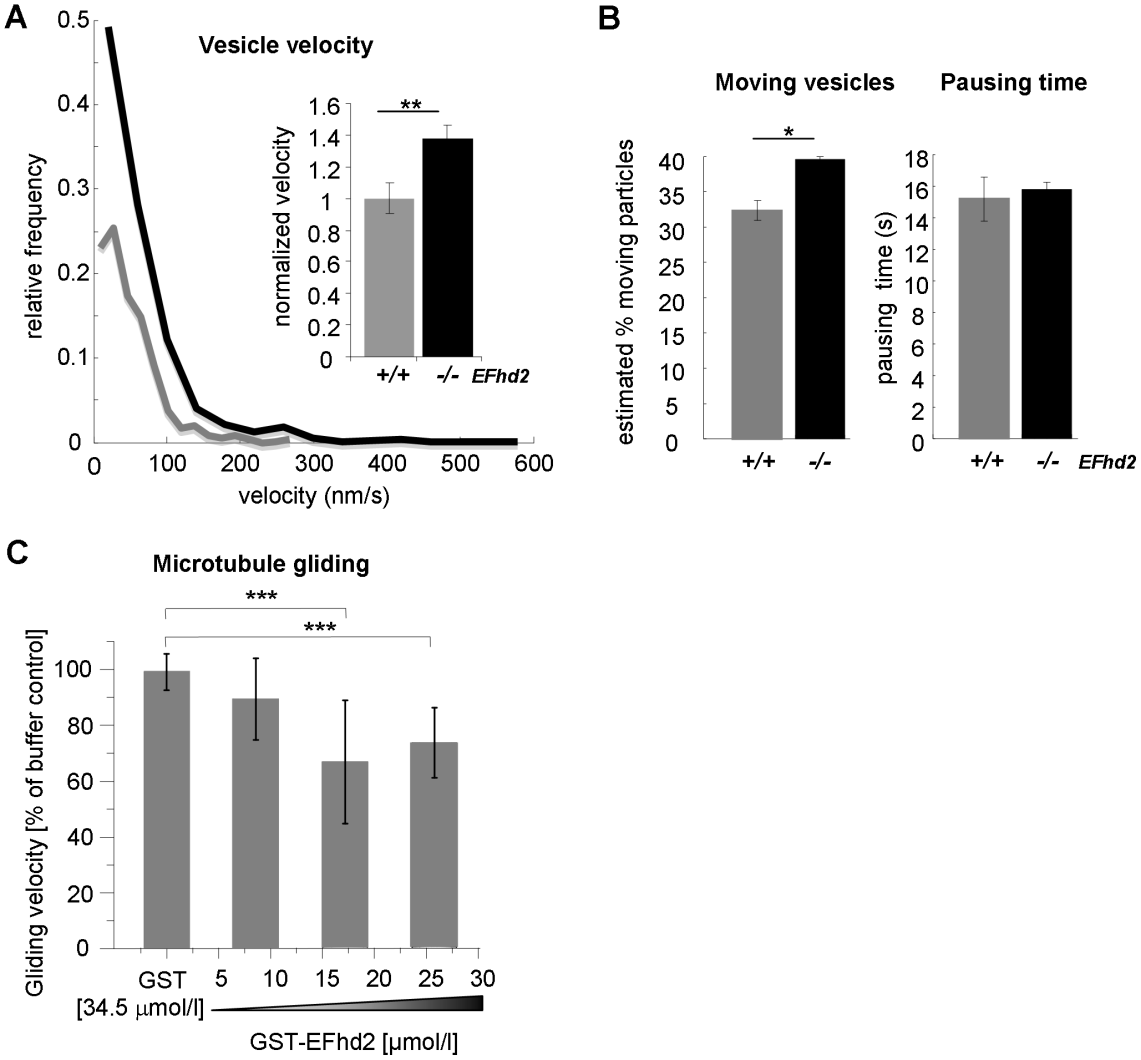
Inhibitory effects of EFhd2 on microtubule motor mediated transport in cellular and cell-free systems. (A) Primary hippocampal neurons of EFhd2^+/+^ and EFhd2^−/−^ mice were transfected to express synaptophysin-EGFP. The velocity of vesicles marked with synaptophysin-EGFP was determined and is plotted both against the relative frequency and as normalized velocity (EFhd2^+/+^ = 1; mean −/+ SEM). The average velocities are 59.98−/+16.76 nm/s (EFhd2^−/−^; 25 kymographs) vs. 47.06−/+12.73 nm/s (EFhd2^+/+^; 10 kymographs) (mean−/+ SD; p = 0.0063, two tailed t-test). Results represent 4 experiments. (*B*) Primary hippocampal neurons of EFhd2^+/+^ and EFhd2^−/−^ mice were transfected to express synaptophysin-EGFP. The number and pausing times of moving vesicles marked with synaptophysin-EGFP were quantified and data of 4 experiments was represented as mean ± SEM (*** *P*<0.001; two tailed student's t-test). (*C*) Purified KIF5A_560_ was coated to glass slides and incubated with polymerized MTs in the absence or presence of GST or increasing amounts GST-EFhd2. The gliding velocity was determined and is shown as % of the buffer control that was set as 100% (corresponding to a gliding velocity of 1.3 um/sec). Data are represented as mean ± SD of five independent experiments. In every experiment, gliding velocities of 10–15 microtubules were measured for each experimental condition. *** *P*<0.001; one way ANOVA followed by a Dunett's multiple comparisons test.

Nevertheless, the distribution of EFhd2 along axons and dendrites in a punctuate fashion (Figs. 4 and 5), and its presence in biochemically isolated synaptosomes without functional effect on synaptic exocytosis or endocytosis (Fig. 6), raised the possibility that EFhd2 may be involved in axonal transport. Therefore, we measured synaptic transport by expressing a synaptophysin-GFP fusion protein, coupled to an automated image acquisition and analysis device [21], in primary hippocampal neurons of EFhd2^+/+^ and EFhd2^−/−^ mice. Interestingly, net axonal transport and percentages of moving particles were significantly increased in the absence of EFhd2, whereas the pausing time was not affected (Fig. 7A, B). Thus, lack of EFhd2 increased the speed of axonal transport.

We next asked, whether EFhd2 decreased axonal transport by inhibition of the plus end microtubule motor protein kinesin [11]. We quantified kinesin activity in a microtubule-gliding assay [24], [31]. In this cell free *in vitro* assay system, purified neuron specific kinesin (KIF5A) was coated on a glass surface, overlaid with polymerized MTs and movement of MTs was analyzed by microscopy [31]. Interestingly, the recombinant GST-EFhd2 fusion protein [2], but not GST alone, inhibited KIF5A mediated MT gliding in a dose-dependent manner (Fig. 7C). These data indicated that kinesin mediated transport was modulated in the presence of EFhd2. We therefore measured the ATPase activity of KIF5A in the absence or presence of recombinant GST-EFhd2. We did not observe a significant inhibition of kinesin mediated ATPase activity by GST-EFhd2 (data not shown). Thus, EFhd2 slowed kinesin mediated MT gliding, which might be a consequence of reduced kinesin-MT interaction.

## Discussion

Strong EFhd2 expression in the brain was detected in the grey matter, including the cortex and hippocampus. Thus, the *lacZ* expression pattern in EFhd2-gene targeted mice and anti-EFhd2 immunohistochemistry outlined a specific EFhd2 expression in the pyramidal layers of the cortex, in the dentate gyrus, and in the CA1-CA2 regions of the hippocampus. These findings are in line with the *in situ* hybridizations described in the Allen brain atlas where areas with a high density of neurons also intensively stain for *efhd2*, whereas regions with fewer neurons also show less *efhd2* expression. Brain regions that mostly consist of white matter (e.g. the corpus callosum) do not show *efhd2* expression (see Allan Brain Atlas, Experiment 69671911). Interestingly, these findings were also confirmed in microarrays from human brain (Allan Brain Atlas, EFhd2 human microarrays).

In the developing embryo, we show for the first time that the EFhd2 protein is expressed in the cortex, hippocampus, and thalamus of E18 brain, indicative of expression of EFhd2 during brain development. EFhd2 was also expressed in primary cortical neurons and became up-regulated during neuronal differentiation in a biphasic manner. This might be an indication that EFhd2 is involved in specific neuronal differentiation processes. Along this line, tau also plays an important physiological role during axon branching and in synaptic plasticity and EFhd2 has been shown to interact with a mutant form of tau [4]. Increased levels of EFhd2 correlated with the presence of an additional band of tau. Thus, a possible interplay between tau and EFhd2 may functionally affect certain specific aspects of neuronal development [12].

To unravel potential functions of EFhd2 in the central nervous system, we studied the intracellular localization of EFhd2. We found EFhd2 expressed in fine granular structures in the neuropil throughout the CNS with prominent expression in regions that contain dense neuronal populations in rodents and human brain (e.g. in the hippocampus), while EFhd2 expression was absent in EFhd2^−/−^ mice. We also found co-labelling of EFhd2 with axonal and dendritic markers in cortical neurons. A co-localization of roughly 40% was observed for EFhd2 with pre-synaptic markers.

Co-staining of EFhd2 with synaptic markers was indicative of a potential role in synaptic transmission. This finding led us to investigate EFhd2 expression in synaptosomes. We detected EFhd2 in biochemically isolated synaptosomes, with the highest abundance in the cytosolic fraction. Other proteins that are predominantly found in the cytosolic fraction, for example amphiphysin, function in synaptic vesicle endocytosis [32]. Analysis of the Drosophila homolog of EFhd2 (DSwip) also revealed a potential role in cell-cell communication: DSwip accumulates transiently in foci at the contact sites of fusing myoblasts and either directly or indirectly regulates calcium-dependent exocytosis of the electron-dense vesicles of the pre-fusion complex in myoblasts [33]. Taking these data into consideration, we next analyzed synaptic exocytosis and endocytosis in EFhd2^−/−^ mice, but did not find any significant difference compared to the control group. This finding might be explained by redundancy of other proteins involved in vesicle exocytosis in the nervous system. For example, even depletion of synaptophysin, a major synaptic vesicle protein involved in endocytosis of synaptic vesicles, does not result in major differences in phenotype or neurotransmitter release [34]–[36]. In line with the proposed function of DSwip, however, the yet to be molecularly defined association of EFhd2 with vesicular structures might be compatible with a function in establishing and breaking down contact between pre- and post-synaptic membrane structures [11], [12], that is, synaptic plasticity.

The distribution of EFhd2 in neurites marked both with tau and MAP2 suggested a universal role in neurites. To our surprise we found an increase in transport velocity and an increase in the number of moving particles in EFhd2^−/−^ mice. The idea of EFhd2 as a regulator of axonal transport is fully compatible with a possible function of EFhd2 in establishing and breaking down contacts between pre- and post-synaptic membrane structures [11]. In addition, the strongest expression of EFhd2 was found in the cytosolic fraction of our biochemically isolated synaptosome preparation. In this fraction, also cytoskeleton markers like β3-tubulin are highly abundant. These data are compatible with EFhd2 being part of a conserved regulatory cytoskeletal network [10], [37].

To address this possibility further, we aimed to identify in a cell free system the proteins that functionally interact with EFhd2. KIF5A-induced MT gliding was decelerated by addition of a GST-EFhd2 fusion protein to this system, indicating a dose-dependent functional interaction between EFhd2 and KIF5A. A potential mechanism of the interference with kinesin activity towards MTs could be inhibition of MT binding to kinesin by EFhd2.

EFhd2 might also exert effects on dynein (MT minus end motor), specifically since differential regulation of dynein and kinesin motor proteins by locally altered concentrations of tau have been described earlier [38]. On the other hand, neither tau-deficient nor tau-overexpressing mice do show alterations of axonal transport *in vivo* [39]. Thus, alternatively and not mutually exclusive, EFhd2 might in neurons also interact with components of the actin cytoskeleton [8], [10], [40], like gelsolin does, which inhibits axonal transport in a Ca^2+^ dependent manner [41].

In our hands, EFhd2^−/−^ mice did not develop any obvious neuronal phenotype. How can this be reconciled with a proposed role for EFhd2 in tauopathies [4], [9], such as Alzheimer's disease [42]? Normal mice do not develop neurodegenerative diseases such as Alzheimer's disease and thus, transgenic mouse models are being used to mimick certain aspects of human neurodegeneration. Hence, it is not surprising that EFhd2^−/−^ mice do not develop an obvious phenotype in that respect. However, the phenotype of induced neurodegeneration, or other neurologic diseases (for review see [7]), upon genetic or environmental challenge, might be modulated in one or the other direction in the absence of EFhd2. For instance, reduction of endogenous tau protein ameliorates onset of disease in an amyloid-beta transgenic mouse model [43]. Alterations in both anterograde as well as retrograde transport are sufficient to induce neurodegeneration [44]. Thus it is tempting to speculate that EFhd2 might be involved in transport of cargo relevant for neurodegenerative diseases, in combination with or without tau. Hence our data will in the future contribute to more specifically testing the hypotheses that EFhd2 is involved 1) in tauopathies [4], [9] with regard to intracellular transport, and 2) in establishment of synaptic plasticity.

Taken together, we reveal here for the first time the wide spread neuronal expression of EFhd2 and propose that EFhd2 is a neuronal protein controlling basal neuronal functions exerted through kinesin.
